# An ‘airy’ cardiac silhouette—pumping in the wrong direction

**DOI:** 10.1007/s12471-022-01750-3

**Published:** 2022-12-12

**Authors:** J. M. Santos, V. D. Neto, E. Correia, J. G. Pereira

**Affiliations:** Department of Cardiology, Tondela-Viseu Hospital Centre, Viseu, Portugal

**Fig. 1:**
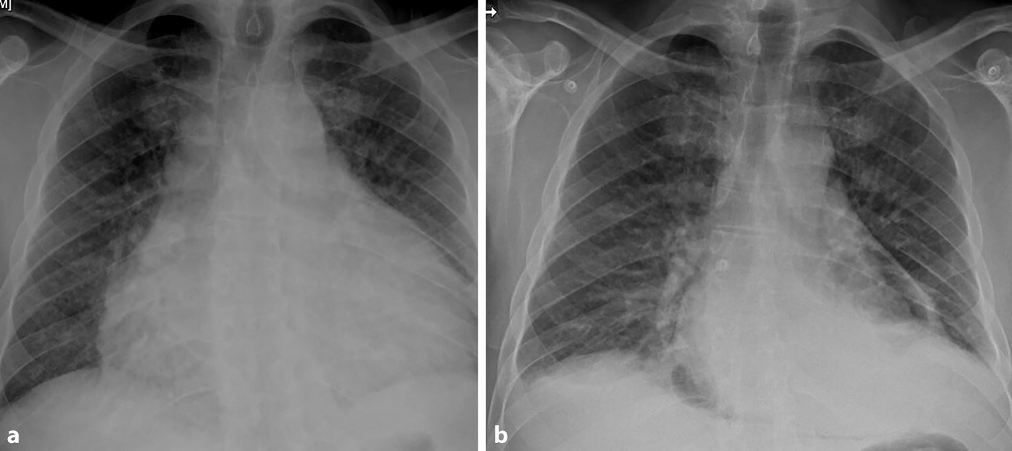
Chest radiographs, **a** obtained at admission showing enlarged cardiac silhouette due to pericardial effusion and **b** obtained 24 h after pericardiocentesis showing iatrogenic pneumopericardium

A 44-year-old man with a past medical history of epilepsy was admitted to the hospital because of dyspnoea. Physical examination revealed hypotension (blood pressure: 88/60 mm Hg), tachycardia (heart rate: 115 bpm), polypnoea, jugular venous distention and muffled heart sounds. Blood analysis showed leucocytosis, neutrophilia and an elevated C‑reactive protein level (12.2 mg/dl). Electrocardiography indicated atrial fibrillation and low voltage on the frontal plane. A chest radiograph revealed an enlarged cardiac silhouette with mild pleural effusion (Fig. 1a). Bedside echocardiography confirmed the presence of severe pericardial effusion with signs of ventricular interdependence. Emergent pericardiocentesis was performed, after which the patient improved haemodynamically.

However, 24 h after the pericardiocentesis, the patient complained of worsening dyspnoea. A repeat chest radiograph showed a pneumopericardium (Fig. 1b), presumably caused by accidental entrapment of air via the pericardial drain during changing of the drainage bag. A conservative approach was decided, and the pneumopericardium resolved spontaneously within one week.

